# Sedentary behavior type matters: compositional analysis of 24-hour movement behaviors and cognitive function in older adults

**DOI:** 10.1186/s44167-026-00105-2

**Published:** 2026-06-16

**Authors:** Xinyu Fan, Yijian Yang, Yang Zhao, Jing Liao

**Affiliations:** 1 Department of Medical Statistics, School of Public Health, Sun Yat-sen University, Zhongshan 2nd Road, Guangzhou, 510080 People’s Republic of China; 2 Department of Sports Science and Physical Education, Chinese University of Hong Kong, Hong Kong, People’s Republic Of China; 3 Intelligent Sensing and Proactive Health Research Center, School of Public Health (Shenzhen), Sun Yat-sen University, Shenzhen, People’s Republic of China

**Keywords:** 24-hour movement behaviors, Older adults, Cognitive function, Mentally sedentary behavior, Compositional data analysis

## Abstract

**Background:**

Sleep, sedentary behaviors (SB) and physical activity are independently associated with cognitive function in older adults, yet the joint relationship of these 24-hour movement behaviors with cognitive function is less well studied. Additionally, the association between SB and cognitive function may differ depending on whether SB is mentally active or inactive. This study aimed to examine the associations between 24-hour movement behavior compositions and cognitive function in older adults, explore the differential associations of different sedentary behavior (SB) types with cognitive function, and assess the predicted cognitive differences associated with time reallocation among these behaviors.

**Methods:**

Data were drawn from 2516 US adults aged ≥ 60 years in the 2011–2014 National Health and Nutrition Examination Survey. Sleep, total SB, light physical activity (LPA), and moderate-to-vigorous physical activity (MVPA) were all assessed via wrist-worn accelerometry. Total SB was disaggregated into TV watching (inactive SB), computer use (active SB), and other SB using individual proportional weights derived from the Global Physical Activity Questionnaire (GPAQ). Cognitive function was evaluated using the Consortium to Establish a Registry for Alzheimer’s Disease Word Learning (memory), Animal Fluency (language), and Digit Symbol Substitution (executive function) tests. Standardized scores were combined into a global cognition score. Weighted compositional linear regression and isotemporal substitution models were applied.

**Results:**

In the 4-component model, greater relative time in MVPA and total SB were positively associated with global cognition (*β* = 0.191 and *β* = 0.260, respectively; both *P* ≤ 0.001), whereas relative sleep and LPA were negatively associated with global cognition. In the 6-component model, disaggregating total SB revealed divergent associations: computer use was positively associated with all cognitive domains (global: *β* = 0.045, *P* < 0.001), while TV watching was negatively associated with global cognition (*β*=−0.050, *P* = 0.047) and language. Notably, neither “other SB” nor sleep remained significantly associated with cognition in this expanded model. Isotemporal substitution analyses indicated that reallocating time to MVPA or computer use was associated with predicted higher global cognition scores, revealing a pronounced asymmetric trajectory where reducing these behaviors predicted steep cognitive declines.

**Conclusions:**

Engaging in MVPA or mentally active SB may help preserve cognitive function in older adults, whereas mentally inactive SB is associated with poorer cognition. Optimizing 24-hour time use by replacing passive sedentary behaviors with MVPA or cognitively engaging activities represents a promising strategy.

**Supplementary Information:**

The online version contains supplementary material available at 10.1186/s44167-026-00105-2.

## Introduction

A growing body of evidence indicates that physical activity, sleep and sedentary behaviors (SB) are independently associated with cognitive function in older adults [1, 2]. Specifically, moderate-to-vigorous physical activity (MVPA) has been consistently associated with better cognitive function [3], and emerging evidence suggests that light physical activity (LPA) may also be positively associated with cognitive outcomes, though findings remain less consistent [4]. Sleep typically shows an inverted U-shaped association with cognition, where both insufficient and excessive sleep duration are associated with poorer cognitive outcomes [5, 6]. In contrast, the relationship between total SB and cognition remains inconclusive, with studies reporting positive, negative, or no associations [7], suggesting that the type of SB may play a key role. However, most existing studies rely on standard regression models that treat 24-hour movement behaviors (sleep, SB, LPA, and MVPA) as isolated exposures, overlooking their fundamental interdependence within a finite 24-hour cycle.

To address this limitation, compositional data analysis (CoDA) has emerged as a robust approach to capture the integrated nature of movement behaviors [8–10]. CoDA deals with co-dependent variables whose sum is a constant value, such as the 1440 min in a 24-hour day allocated to various movement behaviors [11–13]. Several studies have applied CoDA to examine how the composition of 24-hour movement behaviors relates to cognitive function in older adults. Findings from these studies suggest that increased time spent in MVPA was associated with better cognitive function, but inconsistent results for other movement behaviors [14–18]. Notably, one cross-sectional analysis within a longitudinal study reported no significant associations [19, 20], further highlighting the inconsistencies in the current evidence base. These mixed findings, coupled with small sample sizes (typically 400–600 participants) and limited population representativeness, underscore the need for larger, population-based analysis.

Moreover, most previous studies have neglected the mental aspect of SB, which can be either mentally active (e.g., reading, working, using computer) or mentally inactive (e.g., watching TV) [21]. To our knowledge, only one study using CoDA has examined the association between activity domain (e.g., quiet time, social, and screen time) and cognitive function, finding that the domain of behaviors should be considered when supporting cognitive function [22]. Another study explored the interaction between TV viewing and the 24-hour movement behavior composition, but found no significant associations and did not incorporate SB types into the compositional framework [20]. The relationship between SB time and cognitive function appears to be influenced by various factors, including measurement methods of SB (self-reported or device-based), domains of SB, and the specific cognitive domains [23–25]. Within the framework of 24-hour movement behaviors, the nature of SB—whether mentally active or inactive—remains an underexplored but potentially important factor in clarifying its cognitive effects. For older adults with limited mobility who cannot increase PA, determining whether reallocating time from mentally inactive SB to mentally active SB confers cognitive benefits is of high practical value.

The study aimed to examine how the compositions of 24-hour movement behaviors were associated with cognitive function in older adults. These behaviors, which collectively comprise the entire 24-hour cycle, consist of sleep, SB, LPA, and MVPA, with SB further categorized into mentally active and inactive forms. In addition, the study investigated how reallocating time among these behaviors was associated with predicted differences in cognitive function.

## Methods

### Study sample

The present study’s data were drawn from NHANES, a nationally representative sample of the non-institutionalized US population [26]. NHANES study (Protocol #2011-17) was approved by the National Center for Health Statistics Ethics Review Board and all participants provided written informed consent. We combined the 2011–2012 and the 2013–2014 cycles of NHANES for the current cross-sectional analysis. This analysis included 2516 participants aged 60 years and older without cognitive impairment, with complete data on cognitive tests, Global Physical Activity Questionnaire (GPAQ), valid accelerometer records, and all covariates. The flow chart (Fig. S1) is showed in Supplementary Material.

## The 24-hour movement behaviors measurements

NHANES participants were instructed to wear an ActiGraph GT3X+ accelerometer (Actigraph, Pensacola, FL) on their non-dominant wrist for 7 consecutive days (midnight to midnight). Raw acceleration data were processed into Monitor-Independent Motion Summary (MIMS) units using a validated open-source algorithm [27]. The MIMS unit is a summary measure of acceleration used to quantify physical activity, with higher values indicating greater levels of activity. Sleep and wake periods were identified from minute-level MIMS data using the Tudor-Locke algorithm [28]. All minutes within identified sleep periods were classified as sleep.

Within wake periods, minutes were further classified into SB, LPA, and MVPA based on MIMS values. As universally accepted MIMS-based intensity thresholds are currently lacking, we referenced a study that mapped MIMS values to activity counts, the most widely used and validated accelerometer metric [29]. Based on this mapping, minutes with MIMS < 10.558 were classified as SB.

For MVPA, age- and sex-specific thresholds were applied to account for variation in physical capacity across subgroups. Thresholds were derived following the approach developed by Migueles et al. [30], adapted to the present study. Thresholds were estimated using NHANES 2011–2014 participants aged 18–85 years with valid accelerometry data (*n* = 9249; 4416 males, 4833 females), after excluding invalid wear days and days with ≥ 72 min predicted as non-wear. Age- and sex-specific MVPA thresholds (MIMS units) are presented in Supplementary Table S1. Accordingly, a wake minute was classified as MVPA if its MIMS value exceeded the participant’s age- and sex-specific threshold; as LPA if MIMS ≥ 10.558 and ≤ the MVPA threshold.

A day was considered valid if it included at least 1368 min (95% of a day) of wear time and less than 17 h of recorded sleep. Days not meeting these criteria were excluded. Periods of non-wear time were filled in using the individual’s average valid MIMS units for the corresponding time of day, which was calculated based on their remaining valid wear days. Participants who had more than 3 valid days, which had to include at least 2 weekdays and 1 weekend day, were kept for analysis. A previous study found one day of data to be sufficiently stable for population-level analysis, so we averaged multiple days of data into one day [31].

Since the mental aspect of SB cannot be distinguished using accelerometry alone, we used the relative proportion of time reported in GPAQ to break down the accelerometer-derived total SB into TV watching, computer use, and other SB. TV watching and computer use were selected as proxies for mentally inactive and mentally active SB, respectively. Specifically, participants were asked to report their average daily time spent on TV watching (“how many hours per day did you sit and watch TV or videos”) or computer use (“how many hours per day did you use a computer or play computer games outside of school”) over the past 30 days. Although predominantly recreational in this older adult sample, such computer-based activities (e.g., managing personal finances, reading news online, or playing digital games) generally require interactive cognitive engagement, justifying its use as a proxy for mentally active SB.

Response options for these specific behaviors included: “Do not watch/use”, “Less than 1 hour”, “1 hour”, “2 hours”, “3 hours”, “4 hours”, and “5 hours or more”, which correspond to 0, 30, 60, 120, 180, 240, and 300 min, respectively. The proportion of total SB attributed to TV watching and computer use was derived from the ratio of self-reported TV and computer time to total self-reported sitting time. For participants where the sum of self-reported TV and computer time exceeded total reported sitting time (*n* = 343, 13.6%), likely reflecting underreporting of sitting time or overreporting of specific activities, the relative ratio of TV to computer use was preserved, while the combined proportion was capped at the median value observed in the remaining sample (58.3%). The remaining proportion of the accelerometer-derived SB time was classified as “other SB”. This category served as a residual aggregate encompassing all other unmeasured SB, such as reading, socializing, dining, and passive travel. Given the compositional heterogeneity of these activities, inferences regarding the specific cognitive nature of “other SB” were inherently limited.

## Cognitive function measurements

Cognitive assessment in NHANES 2011–2014 included three domains: memory, language fluency, and executive function. Memory was measured using the Consortium to Establish a Registry for Alzheimer’s Disease Word Learning (CERAD W-L) subtest [32]; language fluency by the Animal Fluency Test (AFT) [33]; and executive function by the Digit Symbol Substitution test (DSST) [34].

The CERAD W-L subtest consisted of 3 consecutive learning trials and 1 delayed recall trial. In each learning trial, participants read a list of 10 unrelated words, with the orders of words changed across trials. After completing the AFT and DSST, participants were asked to recall as many words as possible from the initial list. One point was recorded for each correctly recalled word, yielding a total score ranging from 0 to 40.

In the AFT, participants were asked to name as many animals as possible within one minute, with one point recorded per unique animal named. The test emphasizes semantic fluency and general knowledge, minimizing cultural or educational bias. Before the formal test, NHANES participants completed a practice trial by naming three items of clothing. Those unable to complete this task were not administered the full AFT. Scores ranged from 0 upward with no upper limit.

The DSST required participants to copy symbols corresponding to digits 1–9 using a key displayed at the top of the page. Participants had two minutes to fill in symbols across 133 boxes, receiving one point for each correct entry, for a total possible score of 0 to 133.

Raw scores from each cognitive test were standardized into z-scores. A global cognition z-score was calculated by averaging the z-scores of three tests and then dividing by the standard deviation.

## Covariates

Covariates in this study comprised demographic characteristics and some risk factors for dementia [35]. Demographic variables included age (continuous), sex (male or female), education level (under high school, high school or equivalent, above high school), marriage (married/living with partner, widowed/divorced/separated, never married), and ethnicity (non-Hispanic White, non-Hispanic Black, non-Hispanic Asian, Hispanic, or other). The factors associated with dementia risk included obesity (defined as a body mass index [BMI] > 29.9 kg/m^2^), smoking status (yes or no), excessive drinking (defined as an average weekly alcohol consumption of ≥ 15 units for males or ≥ 8 units for females), hypertension (based on self-reported physician diagnosis), diabetes (based on self-reported physician diagnosis), and physical function limitations (defined as any self-reported limitation in daily activities due to physical, mental, or emotional problems). Except for obesity, which was determined via physical measurements, all other covariates were derived from corresponding self-reported questionnaire.

### Statistical analysis

Since log-ratio transformations require strictly positive values, zero values within the movement behavior compositions were first imputed using the log-ratio expectation-maximization algorithm. Descriptive statistics were calculated to summarize participant characteristics; unweighted values were presented to describe the analytical sample, whereas weighted values were utilized to account for the complex, multistage sampling design of NHANES and to produce nationally representative estimates. Because compositional data are subject to a constant sum constraint (i.e., 1440 min per day), central tendencies of the 24-hour movement behaviors were summarized using compositional geometric means [12]. A pairwise log-ratio variance matrix was computed to assess the dispersion of the compositional data, providing estimates such as the variance of ln (Sleep/SB). Lower log-ratio variances indicate greater codependence between the corresponding movement behaviors.

The composition of movement behaviors was expressed as ratios of its parts using isometric log-ratio (ilr) transformations, allowing their appropriate inclusion in linear regression models. All ilr coordinates, along with covariates, were entered into weighted compositional multiple linear regression models to examine associations between 24-hour movement behaviors and both domain-specific and global cognitive function. Ilr coordinates were constructed using sequential binary partition. The first ilr coordinate (corresponding to the *β*_*1*_ coefficient in the regression model, e.g., $\sqrt {\frac{3}{4}} \ln \frac{{x_{1} }}{{\sqrt[3]{{x_{2} \cdot x_{3} \cdot x_{4} }}}}$) represented the relative contribution of one behavior compared to the geometric average of the remaining behaviors [36]. The number of ilr coordinates generated was one fewer than the number of movement behavior components. Because ilr transformations are permutation invariant, the order of behaviors was iteratively rotated in the regression models without affecting the overall model fit [12], allowing the relative association of each movement behavior to be evaluated against the others.

Building on the fitted compositional regression models, isotemporal substitution analysis was applied to model the predicted differences in cognitive function associated with hypothetical time reallocation between movement behaviors [37]. It is important to note that because these analyses are based on cross-sectional data, the estimated reallocations represent hypothetical, associational differences rather than causal effects. Specifically, we examined the associated differences in cognitive scores when reallocating 10 min from one behavior to another, or when distributing time from one behavior proportionally across the remaining behaviors. To illustrate the continuous dose-response trajectories, association curves were plotted to map the predicted changes in cognitive function against progressive reallocation durations. The regression model was applied iteratively in 1-minute increments, up to a maximum reallocation of 60 min. This 60-minute boundary encompasses clinically meaningful behavioral changes while remaining plausible within typical daily constraints. Crucially, to strictly maintain the non-negativity constraint of compositional data, the maximum duration of time subtracted from any given behavior was capped at its respective geometric mean (i.e., time reductions ranged from 0 to 60 min, but could never exceed the baseline geometric mean of the specific activity being displaced).

All statistical analyses were performed using R version 4.4.0 (R Core Team, Vienna, Austria). Zero-value imputation was conducted using the “zCompositions” package [38]; compositional data transformations and sequential binary partitioning were implemented using the “compositions” packages [39]; and survey-weighted regression models were fitted using the “survey” package [40]. Statistical significance was determined using two-tailed hypothesis testing with an alpha level of 0.05.

## Results

### Descriptive characteristics

The analytical sample comprised 2516 older adults, representing approximately 45.7 million U.S. adults. The mean age of the unweighted sample was 70.3 years, corresponding to a weighted mean age of 69.9 years. The sample exhibited a relatively balanced sex distribution (weighted: 55.0% female). After accounting for survey weights, the majority of the represented population were non-Hispanic White (79.4%), were married or living with a partner (64.4%), and had an education level above high school (61.4%). Detailed unweighted and weighted participant characteristics are presented in Table 1.

**Table 1:** Characteristics of study population aged 60 years and over from the NHANES 2011–2014

Characteristics	Unweighted (*n*=2516)^a^	Weighted (*n*=45700906)^b^
Age	70.3 (8.0)	69.9 (0.2)
*Sex*		
Male	1213 (48.2%)	45.0% (1.1%)
Female	1303 (51.8%)	55.0% (1.1%)
*Education level*		
Under high school	648 (25.8%)	16.4% (1.5%)
High school or equivalent	598 (23.8%)	22.2% (1.3%)
Above high school	1270 (50.5%)	61.4% (1.7%)
*Marriage*		
Married/living with partner	1441 (57.3%)	64.4% (1.1%)
Widowed/divorced/separated	926 (36.8%)	31.1% (1.0%)
Never married	149 (5.9%)	4.5% (0.5%)
*Ethnicity*		
Non-Hispanic		
White	1206 (47.9%)	79.4% (1.9%)
Black	613 (24.4%)	8.7% (1.2%)
Asian	181 (7.2%)	2.9% (0.5%)
Hispanic	481 (19.1%)	7.2% (1.1%)
Other ethnicity	35 (1.4%)	1.8% (0.6%)
*Obesity*		
No	1540 (61.2%)	60.7% (1.4%)
Yes	976 (38.8%)	39.3% (1.4%)
*Smoking status*		
No	2202 (87.5%)	89.3% (0.7%)
Yes	314 (12.5%)	10.7% (0.7%)
*Excessive drinking*		
No	2390 (95%)	93.3% (0.9%)
Yes	126 (5.0%)	6.7% (0.9%)
*Hypertension*		
No	924 (36.7%)	40.2% (1.3%)
Yes	1592 (63.3%)	59.8% (1.3%)
*Diabetes*		
No	1904 (75.7%)	79.6% (1.1%)
Yes	612 (24.3%)	20.4% (1.1%)
*Physical function limitations*		
No	1441 (57.3%)	62.2% (1.6%)
Yes	1075 (42.7%)	37.8% (1.6%)
Sleep (min/day)	456.4 (96)	464.2 (2.9)
Total SB (min/day)	550.1 (128.7)	545.5 (3.9)
LPA (min/day)	406.8 (127.5)	402.8 (3.9)
MVPA (min/day)	26.8 (30.9)	27.6 (0.8)
TV watching (min/day)	254.3 (140.5)	249.2 (5.9)
Computer use (min/day)	65.3 (86.9)	75.3 (2.8)
Other SB (min/day)	230.5 (145.3)	221.0 (4.1)
CERAD word learning subtest score^c^	24.6 (6.7)	25.8 (0.3)
Animal fluency test score^d^	16.5 (5.6)	18.1 (0.2)
Digit symbol substitution test score^e^	44.8 (18.5)	51.3 (0.6)
Cognitive test total scores	85.9 (26.3)	95.2 (0.9)

NHANES: National health and nutrition examination survey; SB: sedentary behaviors; LPA: light physical activity; MVPA: moderate-to-vigorous physical activity

^a^The unweighted sample used mean (standard deviation) for continuous variables and frequency (percentage) for categorical variables

^b^The weighted sample used mean (standard error) for continuous variables and proportion (standard error) for categorical variables

^c^The test score ranges from 0 to 40

^d^The test score ranges from 0 and above (name as many words as you can in 1 min)

^e^The test score ranges from 0 to 133

Geometric means (in minutes and percentages) and pairwise log-ratio variances for the 24-hour movement behaviors are presented in Table 2. In the 4-component model, the largest proportion of daily time was spent in total SB (38.4% or 553.09 min), followed by sleep (32.9% or 473.52 min), LPA (27.6% or 397.80 min), and MVPA, which accounted for the smallest percentage (1.1% or 15.60 min). The lowest log-ratio variance, which means the most interdependence and ability to switch behaviors, was found between total SB and sleep (0.12). Conversely, the highest variances were found between MVPA and total SB (2.06), followed by MVPA and sleep (1.81).

**Table 2:** Geometric mean and pairwise log-ratio variance matrix for 24-hour movement behaviors (weighted)

Indicators	LPA	MVPA	Sleep	Total SB		
				TV watching	Computer use	Other SB
Geometric mean, minutes (%)	397.80 (27.6)	15.60 (1.1)	473.52 (32.9)	553.09 (38.4)		
LPA	0	1.32	0.23	0.28		
MVPA	1.32	0	1.81	2.06		
Sleep	0.23	1.81	0	0.12		
Total SB	0.28	2.06	0.12	0		
Geometric mean, minutes (%)	482.59 (33.5)	18.97 (1.3)	574.45 (39.9)	248.54 (17.3)	7.90 (0.5)	107.55 (7.5)
LPA	0	1.30	0.23	1.09	15.40	9.05
MVPA	1.30	0	1.79	2.86	16.29	10.81
Sleep	0.23	1.79	0	0.88	15.50	8.88
TV watching	1.09	2.86	0.88	0	17.11	11.12
Computer use	15.40	16.29	15.50	17.11	0	25.67
Other SB	9.05	10.81	8.88	11.12	25.67	0

SB: sedentary behavior; LPA: light physical activity; MVPA: moderate-to-vigorous physical activity. All results were weighted to account for the complex sampling design of the NHANES. The total duration of all movements is 1440 min–24 h. Sedentary behaviors have been classified into three categories: TV watching, computer use and other sedentary behaviors

In the 6-component model, where total SB was disaggregated, the highest geometric proportion of time was allocated to sleep (39.9% or 574.45 min), followed by LPA (33.5% or 482.59 min), TV watching (17.3% or 248.54 min), other SB (7.5% or 107.55 min), MVPA (1.3% or 18.97 min), and computer use (0.5% or 7.90 min). Regarding pairwise dispersions, LPA and sleep exhibited the lowest log-ratio variance (0.23), suggesting high codependency. In contrast, computer use demonstrated the lowest interdependence with other behaviors, exhibiting the highest log-ratio variances particularly with other SB (25.67) and TV watching (17.11).

## Compositional linear regression of 24-hour movement behaviors on cognitive function

The results of the compositional multiple linear regression examining the associations between 24-hour movement behaviors and cognitive function are presented in Table 3. Overall, the movement behavior compositions were significantly associated with all cognitive outcomes (model *P* < 0.001 for all domains).

**Table 3:** Compositional linear regression of 24-hour movement behaviors on cognitive function (weighted)

Movement behaviors	Memory		Language		Executive function		Global cognition	
	β	*P*	β	*P*	β	*P*	β	*P*
LPA	−0.105	0.106	−0.086	0.321	**−0.319**	**0.001**	**−0.209**	**0.008**
MVPA	**0.142**	**< 0.001**	**0.115**	**0.005**	**0.210**	**< 0.001**	**0.191**	**< 0.001**
Sleep	**−0.256**	**0.002**	**−0.203**	**0.038**	**−0.134**	**0.042**	**−0.242**	**0.001**
Total SB	**0.219**	**0.007**	0.174	0.054	**0.243**	**0.003**	**0.260**	**0.001**
LPA	−0.013	0.810	−0.004	0.957	**−0.222**	**0.005**	−0.098	0.125
MVPA	**0.108**	**< 0.001**	**0.086**	**0.017**	**0.173**	**< 0.001**	**0.150**	**< 0.001**
Sleep	−0.092	0.101	−0.042	0.559	0.0003	0.996	−0.055	0.311
TV watching	−0.044	0.075	**−0.076**	**0.029**	−0.003	0.872	**−0.050**	**0.047**
Computer use	**0.039**	**< 0.001**	**0.027**	**< 0.001**	**0.044**	**< 0.001**	**0.045**	**< 0.001**
Other SB	0.002	0.796	0.010	0.230	0.008	0.221	0.008	0.146

SB: sedentary behavior; LPA: light physical activity; MVPA: moderate-to-vigorous physical activity

The model was weighted based on the complex sampling design of NHANES and adjusted for age, sex, education level, marriage, ethnicity, obesity, smoking status, excessive drinking, hypertension, diabetes, and physical function limitations

The significant results were bold. The significance of *β* was assessed using the Wald test

In the 4-component model, greater relative time spent in MVPA was significantly and positively associated with all cognitive outcomes (memory, language, executive function, and global cognition). Conversely, relative time spent in sleep was significantly and negatively associated with all cognitive outcomes. Total SB, relative to other behaviors, was positively associated with memory, executive function, and global cognition, though its association with language did not reach statistical significance (*β* = 0.174, *P* = 0.054). Greater relative time in LPA was negatively associated with both executive function and global cognition.

In the 6-component model, total SB was disaggregated to explore the differential associations of specific sedentary subtypes. Greater relative time spent in computer use was significantly and positively associated with all domains of cognitive function (memory: *β* = 0.039; language: *β* = 0.027; executive function: *β* = 0.044; global cognition: *β* = 0.045). In contrast, relative time spent watching TV was significantly and negatively associated with language (*β*=-0.076, *P* = 0.029) and global cognition (*β*=-0.050, *P* = 0.047). Notably, relative time spent in other SB was not significantly associated with any cognitive outcome. Furthermore, the significant negative associations for sleep observed in the 4-component model were completely attenuated and became non-significant across all cognitive domains in this expanded model. In this 6-component composition, MVPA remained positively associated with all cognitive outcomes, whereas LPA remained negatively associated only with executive function.

### Compositional isotemporal substitution model on cognitive function

**Fig. 1:**
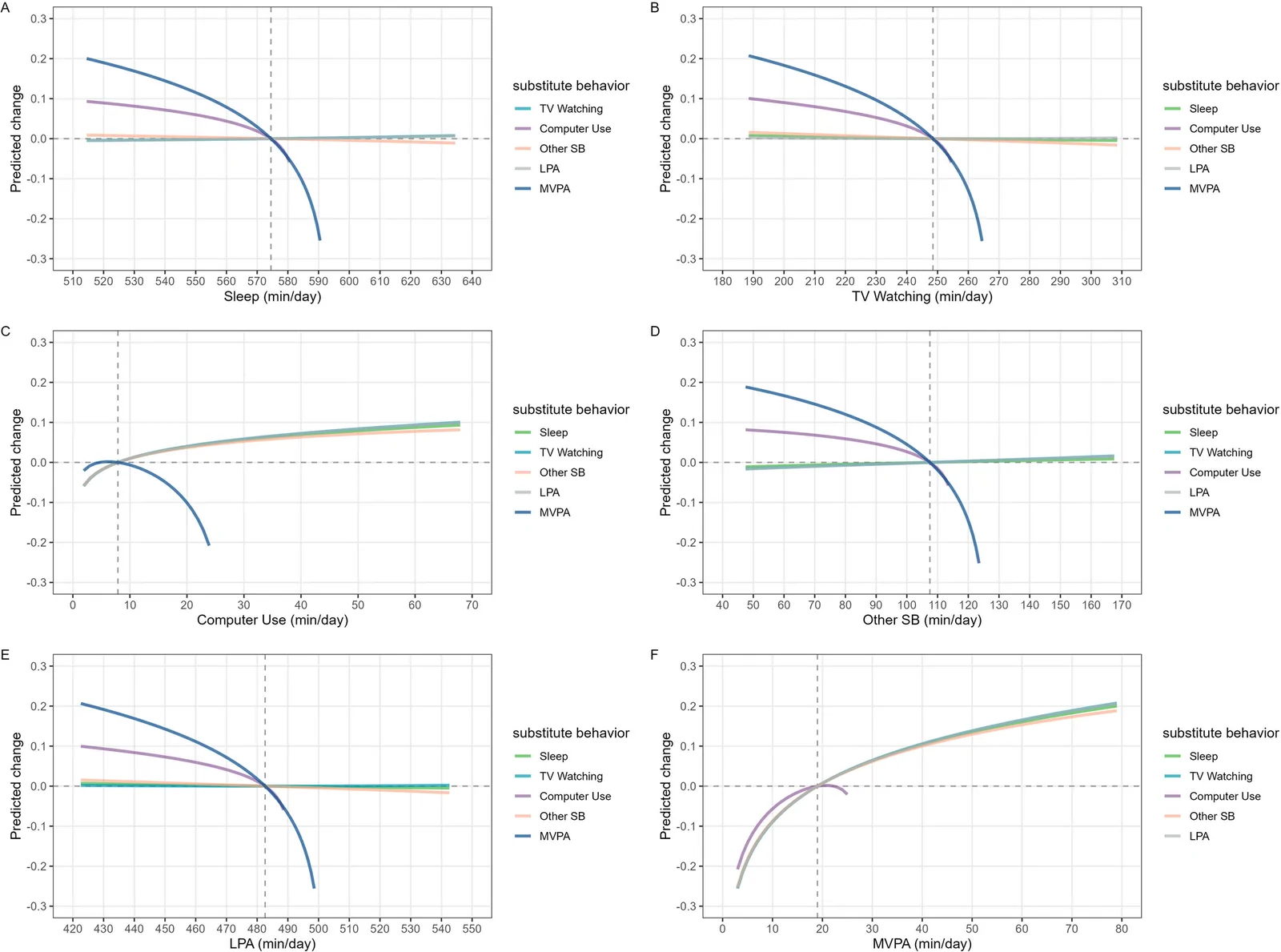
Predicted change in global cognition with hypothetical time reallocation from one movement behavior to another.* Note*: SB: sedentary behavior; LPA: light physical activity; MVPA: moderate-to-vigorous physical activity. The dashed lines represent the geometric means of each behavior among the study participants. The x-axis represents the increase or decrease in the duration of a specific primary behavior, while the other five behaviors increase or decrease the time allocated to that behavior by a proportion relative to their original geometric means. The y-axis represents the predicted change in global cognitive function scores relative to baseline. The curves reflect the effect trajectories of the time allocated to other individual behaviors, as shown in the legend. The model was weighted based on the complex sampling design of NHANES and adjusted for age, sex, education level, marriage, ethnicity, obesity, smoking status, excessive drinking, hypertension, diabetes, and physical function limitations

**Fig. 2:**
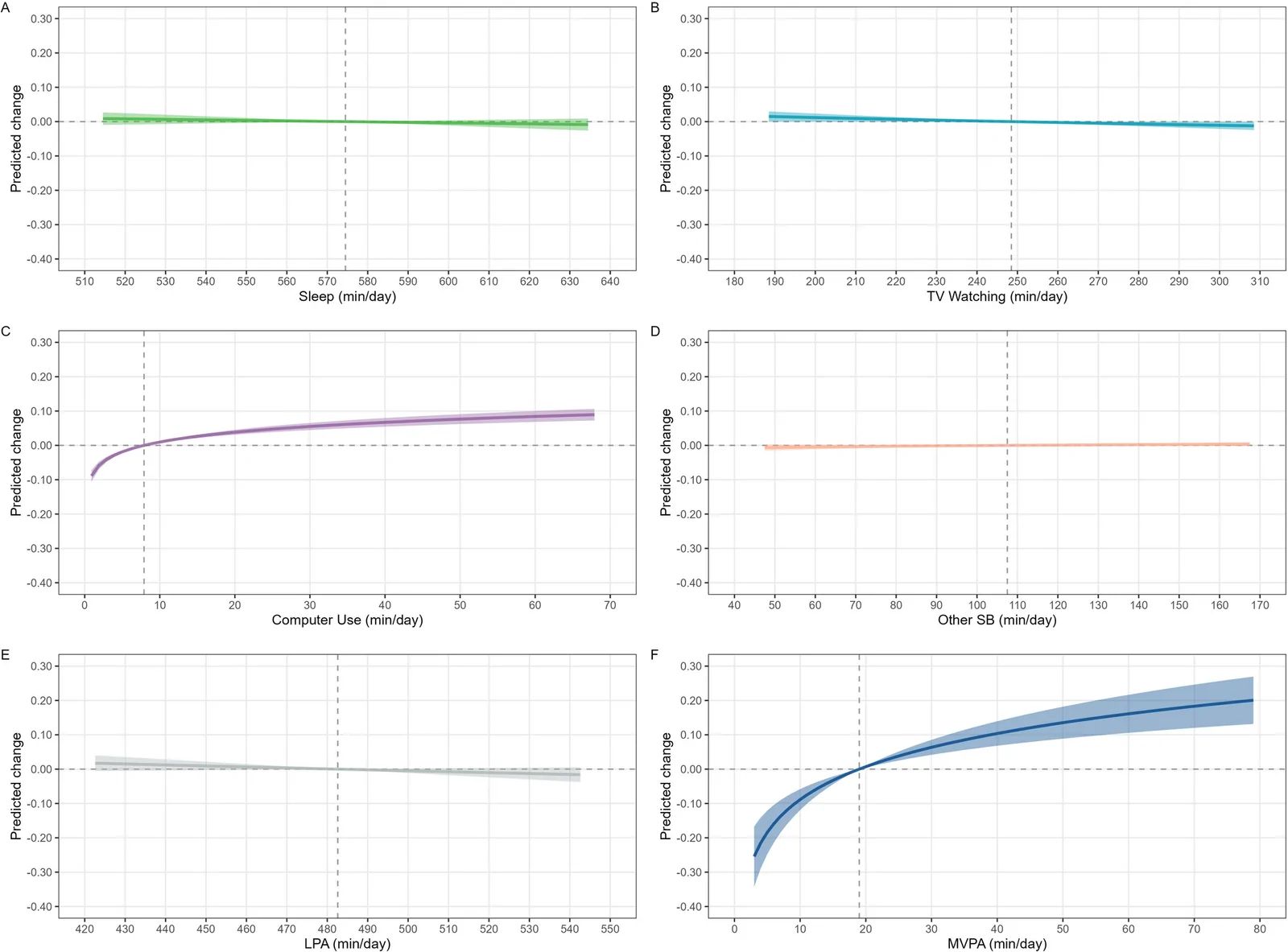
Predicted change of global cognition for reallocating hypothetical time between one and the remaining behaviors.* Note*: SB: sedentary behavior; LPA: light physical activity; MVPA: moderate-to-vigorous physical activity. The dashed lines represent the geometric means of each behavior among the study participants. The x-axis represents the increase or decrease in the duration of a specific primary behavior, while the time allocated to the remaining behaviors increases or decreases in proportion to their original geometric means. The y-axis represents the predicted change in global cognitive function scores relative to baseline. The curves reflect the effect trajectories of providing substitute time on the remaining behaviors. The model was weighted based on the complex sampling design of NHANES and adjusted for age, sex, education level, marriage, ethnicity, obesity, smoking status, excessive drinking, hypertension, diabetes, and physical function limitations

Figure 1 illustrates the predicted continuous dose-response trajectories for global cognition associated with one-to-one time reallocations between different movement behaviors (e.g., substituting 10 min/day of sleep with 10 min/day of MVPA). Figure 2 presents the predicted changes in global cognition associated with one-to-many time reallocations (e.g., displacing 10 min/day of sleep and proportionally distributing that time across all remaining behaviors). The detailed point estimates and 95% confidence intervals (CIs) for 10-minute reallocations across all movement behaviors and cognitive outcomes are provided in Tables S2 and S3 (see Supplementary).

Consistent with the compositional linear regression findings, both Figs. 1 and 2 visually confirm that reallocating time from other behaviors into MVPA or computer use was associated with predicted higher global cognition scores. Conversely, displacing MVPA or computer use with any other behavior was associated with notably poorer predicted cognition. For instance, in Fig. 1, reallocating 10 min/day from sleep to computer use or MVPA yielded predicted increases in global cognition (+ 0.034, 95% CI 0.027 to 0.041 and + 0.059, 95% CI 0.040 to 0.077, respectively). In contrast, replacing sleep with other SB or TV watching showed minimal or no associated cognitive benefits, reflecting their non-significant or negative regression coefficients.

Furthermore, the curves in both figures highlight a pronounced asymmetric association regarding MVPA and computer use. Because the baseline geometric means for these behaviors were relatively small (18.97 min and 7.90 min, respectively), the models predicted a steeper cognitive decline when reducing their time (moving left of the dashed geometric mean lines) compared to the magnitude of cognitive benefit when increasing their time (moving right of the dashed lines). For example, as shown in Fig. 2, reallocating 10 min/day from the remaining movement behaviors to MVPA was associated with a modest expected increase in global cognition (+ 0.059, 95% CI 0.039 to 0.079). But the opposite change, reducing MVPA by 10 min, was linked to a much bigger expected decrease in global cognition (−0.103, 95% CI −0.139 to −0.068). A similar, highly sensitive asymmetric drop was observed when computer use was reduced toward zero.

## Discussion

This study examined the associations between the composition of 24-hour movement behaviors and cognitive function in older adults using CoDA, with particular attention to the cognitive nature of SB. In the 4-component model, greater time spent in MVPA and total SB, relative to other behaviors, was associated with better cognitive function, whereas more relative time spent in sleep and LPA was negatively associated with specific cognitive outcomes. Regarding the nature of SB in the 6-component model, time spent in mentally active SB (computer use) was significantly associated with better cognitive function, while time spent in mentally inactive SB (TV watching) was significantly negatively associated with language and global cognition. Isotemporal reallocations modeled that replacing other movement behaviors with MVPA or computer use was associated with predicted higher cognitive scores. Additionally, an asymmetric relationship was observed between changes in MVPA (or computer use) and global cognition.

Unlike previous studies examining MVPA, LPA, SB, and sleep as independent cognitive predictors, we employed a CoDA approach to evaluate their joint association within 24-hour time-use compositions. Consistent with prior research [14, 18, 41], MVPA was positively associated with cognitive function across all domains. MVPA promotes cardiovascular health, enhances cerebral blood flow, and stimulates neurobiological pathways, including the upregulation of BDNF and hippocampal neurogenesis, which are integral to cognitive maintenance [42]. These intensity-dependent mechanisms are not sufficiently activated by LPA [43]. In the CoDA framework, the negative coefficient for LPA reflects a compositional trade-off: individuals who allocate a disproportionately large share of their day to LPA necessarily spend less time in MVPA and cognitively engaging sedentary activities, both of which showed positive associations with cognitive function in this study. Elevated relative LPA time may therefore indicate a ceiling on physical capacity rather than an active behavioral preference, raising the possibility of a selection effect whereby older adults with pre-existing cognitive limitations or functional constraints are capable only of light-intensity movement—a direction of causality that cannot be excluded in this cross-sectional study. Prior studies have similarly reported null or positive associations between LPA and cognitive function in older adults [14, 44]. However, the evidence for cognitive benefits of LPA remains insufficient, indicating that the intensity of movement rather than light activity per se may be the more important factor for cognitive aging.

The relationship between sleep and cognition in older adults is well characterized as non-linear, with both insufficient and excessive sleep associated with poorer cognitive outcomes, and an optimal window typically reported around 7–8 h [5, 6]. The geometric mean sleep duration in the present sample (approximately 7.9 h) falls within this range, suggesting that the sample as a whole was not sleep-deprived. A negative association for sleep may indicate that individuals with relatively greater sleep duration, particularly those exceeding recommended range, allocate less time to MVPA and other cognitively stimulating waking activities. Because these behaviors were positively associated with cognitive function in the present study, the resulting redistribution of time within the 24-hour composition may be associated with poorer cognitive outcomes. Furthermore, longer time spent in bed may not necessarily indicate more restorative sleep in older adults. Due to age-related sleep fragmentation, extended time in bed may include substantial periods of wakefulness, potentially limiting time available for cognitively stimulating activities during waking hours [45]. Notably, this negative association for sleep was attenuated in the 6-component model, suggesting that the cognitive associations of specific SB types (e.g., computer use) largely absorbed the variance previously captured by relative sleep time.

Total SB showed a positive association with cognitive function relative to other behaviors, which may initially appear counterintuitive. This finding is similar to the prior CoDA studies and a large multi-cohort analysis, which have reported null or positive associations between total SB and cognitive outcomes in older adults [14, 20, 24, 46]. Within the CoDA framework, the *β* coefficient for total SB is the association with cognitive function relative to the geometric mean of all remaining components (sleep, LPA and MVPA combined) simultaneously and not a contrast with any single behavior. The direction of this net effect is therefore governed by the collective influence of all comparator behaviors. In our sample, sleep demonstrated the strongest negative associations with cognition across all domains and accounted for the largest proportion of daily time (excluding SB), while LPA also exhibited negative associations with executive function and global cognition. In contrast, MVPA demonstrated positive associations but constituted only 1.1% of daily time. Accordingly, the positive coefficient for total SB plausibly reflects the net compositional balance, whereby individuals spending relatively more time on SB spend less time on sleep and LPA (both negatively associated with cognition in this composition), such that the overall compositional trade-off is cognitively favorable. Treating total SB as a homogeneous construct, however, risks obscuring behavior-specific effects [22, 47], and the fuller picture emerges only upon disaggregation.

Disaggregating total SB in the 6-component model revealed divergent associations across SB subtypes. Computer use, which is a mentally active form of SB, was positively associated with cognitive function across all domains, aligning with previous protective associations of mentally engaging SB [47]. The cognitive disuse atrophy theory offers a plausible explanation [48]: mentally active SB engages repetitive cognitive processing, supporting cognitive maintenance. Physiologically, cognitively demanding activities increase cerebral blood flow and metabolism, both linked to enhanced cognitive performance [49].

Conversely, consistent with prior research [50], TV watching (mentally inactive SB) was negatively associated with language and global cognition, likely due to its predominantly one-way information flow lacking interactive cognitive engagement that activates language-processing brain areas. These findings collectively underscore the importance of SB type when examining its cognitive associations, and reinforce the value of a compositional analytical approach that accounts for the inherent co-dependence of 24-hour movement behaviors. Nevertheless, the cross-sectional design precludes causal inference, and reverse causality cannot be excluded: individuals with pre-existing cognitive decline may be more likely to disengage from cognitively demanding activities and accumulate passive sedentary time [51]. Future longitudinal studies are warranted to establish the directionality of these associations.

Maintaining or increasing time spent in MVPA or mentally active SB appears critical for supporting cognitive function in older adults. Building on the observed associations, an asymmetrical pattern emerged from the isotemporal substitution models: reallocating time to MVPA or computer use resulted in smaller predicted cognitive improvements than the pronounced predicted cognitive declines observed when time was subtracted from these behaviors. While this asymmetry may be partially attributed to their small baseline geometric means, the findings underscore the importance of preserving even short bouts of MVPA. This is consistent with a prior review indicating that brief episodes of MVPA can confer health benefits for older adults, even if they fall short of the recommended 150 min per week [52]. For SB, limiting total sedentary time and reallocating it toward physical activity has generally been linked to better cognitive outcomes [53]. However, mentally active SB, such as computer use or reading, may also positively contribute to cognitive health [21]. A major limitation of much of the existing literature is that physical activity, SB, and sleep have often been analyzed in isolation, rather than as co-dependent components of a 24-hour time-use composition. From a public health perspective, we suggest a hierarchy of behavioral recommendations: increasing MVPA should remain the primary target for cognitive health across all age groups. However, for older adults with mobility limitations for whom increasing MVPA is not feasible, substituting mentally inactive SB with cognitively engaging activities (e.g., computer use) represents a viable complementary strategy. Moreover, prolonged SB has detrimental effects on physical health regardless of cognitive engagement, reinforcing the need for public health guidelines to consider the combined cognitive and physical impacts of time reallocation across all movement behaviors.

One of the strengths of this study is the measurement of 24-hour movement behaviors using an objective 4-component framework (accelerometry for sleep, total SB, LPA, and MVPA), complemented by an individualized proportional allocation method to disaggregate SB types. We conducted an in-depth analysis of the associations between 24-hour movement behaviors and cognitive function using a CoDA approach, and considering the nature of SB within the 24-hour movement behaviors framework. However, there are several limitations. First, the cross-sectional design of this study limits causal inference. Each participant provided activity data for 3–7 days, and the timing of data collection varied across participants, thereby restricting the generalizability of the findings. It is also possible that individuals with better cognitive function are more likely to engage in MVPA and mentally active SB. Longitudinal randomized controlled trials are needed to explore the long-term effects of altering the composition of movement behaviors on cognitive function. Second, the accelerometer algorithm may misclassify some SB as sleep [27, 29], and the self-reported time of specific SB may be affected by reporting bias. Third, the disaggregation of total SB into subtypes relied on GPAQ response options measured on an ordinal scale. Although midpoint values were used to derive proportional weights, which linked absolute time estimates to the objective accelerometry data, this method still has some measurement imprecision built in. Furthermore, since the measurement was limited to TV watching and computer use, it may affect the observed associations between movement behaviors and cognitive function, as unspecified mentally active SB could confound the relationships between other SB types and cognition. Future research should aim to more precisely measure the composition of 24-hour movement behaviors using both sensitive wearable devices and validated context-specific scales.

## Conclusions

In this study, greater relative time spent in MVPA and mentally active SB (e.g., computer use) was significantly associated with better cognitive function in older adults, whereas mentally inactive SB (e.g., TV watching) was negatively associated with cognition. Our findings emphasize that sedentary behavior is not a homogeneous construct, and its cognitive nature profoundly matters. While preserving or increasing MVPA remains the primary behavioral target for cognitive health, substituting passive, mentally inactive sedentary time with cognitively engaging activities represents a viable complementary strategy. For older adults, optimizing the composition of 24-hour movement behaviors, like preserving time for brisk physical activity or engaging in cognitively stimulating tasks (e.g., reading or computer use) instead of passive screen time, may be a highly promising approach to support cognitive maintenance.

## Supplementary Information

Below is the link to the electronic supplementary material.

Supplementary Material 1

## Data Availability

The NHANES datasets analyzed during the current study are freely and publicly available from the NHANES webpages (https://www.cdc.gov/nchs/nhanes/index.htm) operated and maintained by the Centers for Disease Control and Prevention. The main webpage provides access to the datasets, documentation and codebooks.

## Abbreviations

AFT: Animal fluency test
CERAD W-L: Consortium to establish a registry for Alzheimer’s disease word learning
CoDA: Compositional data analysis
DSST: Digit symbol substitution test
Ilr: Isometric log ratio
LPA: Light physical activity
MVPA: Moderate-to-vigorous physical activity
NHANES: National health and nutrition examination survey
SB: Sedentary behaviors
