# Supplementary material for: Sedentary behavior type matters: compositional analysis of 24-hour movement behaviors and cognitive function in older adults
Source: J Act Sedentary Sleep Behav. 2026 Jun 16;5:15. doi: 10.1186/s44167-026-00105-2 (PMC13501802; doi:10.1186/s44167-026-00105-2)
Supplement: Supplementary file 1 [file 44167_2026_105_MOESM1_ESM.docx]

**Figure S1. Flow chart**

**Table S1. Age- and sex-specified MVPA thresholds (expressed as MIMS units)**

| **Age (year)** | **Male** | **Female** |  | **Age (year)** | **Male** | **Female** |
| --- | --- | --- | --- | --- | --- | --- |
| 18 | 49.996 | 46.757 |  | 52 | 42.487 | 41.137 |
| 19 | 49.772 | 46.663 |  | 53 | 42.033 | 40.803 |
| 20 | 49.549 | 46.569 |  | 54 | 41.570 | 40.466 |
| 21 | 49.328 | 46.474 |  | 55 | 41.103 | 40.126 |
| 22 | 49.111 | 46.379 |  | 56 | 40.636 | 39.781 |
| 23 | 48.900 | 46.283 |  | 57 | 40.170 | 39.431 |
| 24 | 48.695 | 46.186 |  | 58 | 39.708 | 39.075 |
| 25 | 48.497 | 46.088 |  | 59 | 39.251 | 38.713 |
| 26 | 48.307 | 45.988 |  | 60 | 38.798 | 38.345 |
| 27 | 48.124 | 45.887 |  | 61 | 38.348 | 37.970 |
| 28 | 47.949 | 45.784 |  | 62 | 37.898 | 37.588 |
| 29 | 47.784 | 45.679 |  | 63 | 37.445 | 37.201 |
| 30 | 47.629 | 45.573 |  | 64 | 36.989 | 36.807 |
| 31 | 47.484 | 45.465 |  | 65 | 36.530 | 36.407 |
| 32 | 47.348 | 45.356 |  | 66 | 36.067 | 36.002 |
| 33 | 47.219 | 45.244 |  | 67 | 35.600 | 35.591 |
| 34 | 47.096 | 45.131 |  | 68 | 35.128 | 35.175 |
| 35 | 46.977 | 45.016 |  | 69 | 34.652 | 34.757 |
| 36 | 46.857 | 44.898 |  | 70 | 34.170 | 34.338 |
| 37 | 46.733 | 44.773 |  | 71 | 33.686 | 33.919 |
| 38 | 46.601 | 44.640 |  | 72 | 33.201 | 33.501 |
| 39 | 46.458 | 44.496 |  | 73 | 32.718 | 33.084 |
| 40 | 46.300 | 44.339 |  | 74 | 32.237 | 32.667 |
| 41 | 46.125 | 44.166 |  | 75 | 31.759 | 32.251 |
| 42 | 45.926 | 43.974 |  | 76 | 31.285 | 31.836 |
| 43 | 45.703 | 43.764 |  | 77 | 30.816 | 31.423 |
| 44 | 45.450 | 43.533 |  | 78 | 30.354 | 31.011 |
| 45 | 45.168 | 43.283 |  | 79 | 29.897 | 30.602 |
| 46 | 44.858 | 43.014 |  | 80 | 29.447 | 30.193 |
| 47 | 44.520 | 42.727 |  | 81 | 29.003 | 29.785 |
| 48 | 44.155 | 42.427 |  | 82 | 28.565 | 29.380 |
| 49 | 43.768 | 42.115 |  | 83 | 28.133 | 28.977 |
| 50 | 43.359 | 41.794 |  | 84 | 27.708 | 28.577 |
| 51 | 42.931 | 41.467 |  | 85 | 27.288 | 28.182 |

*Note.* MVPA: moderate-to-vigorous physical activity; MIMS: Monitor-Independent Movement Summary.

**Table S2. Predicted change values of cognitive function for reallocating 10-min between movement behaviors**

| Outcome | Movements | Sleep↑ | TV Watching↑ | Use Computer↑ | Other SB↑ | LPA↑ | MVPA↑ |
| --- | --- | --- | --- | --- | --- | --- | --- |
| Memory | Sleep**↓** | - | -0.0001 (-0.003, 0.003) | **0.030 (0.019, 0.042)** | 0.002 (-0.001, 0.004) | 0.001 (-0.003, 0.005) | **0.043 (0.026, 0.060)** |
|  | TV Watching**↓** | 0.0002 (-0.003, 0.003) | - | **0.031 (0.019, 0.042)** | 0.002 (-0.0003, 0.004) | 0.001 (-0.001, 0.004) | **0.043 (0.026, 0.061)** |
|  | Use Computer**↓** | N/A | N/A | - | N/A | N/A | N/A |
|  | Other SB**↓** | -0.002 (-0.004, 0.001) | -0.002 (-0.004, 0.0003) | **0.029 (0.018, 0.039)** | - | -0.0004 (-0.003, 0.002) | **0.042 (0.024, 0.059)** |
|  | LPA**↓** | -0.001 (-0.005, 0.003) | -0.001 (-0.004, 0.001) | **0.029 (0.018, 0.040)** | 0.0004 (-0.002, 0.003) | - | **0.042 (0.023, 0.061)** |
|  | MVPA**↓** | **-0.075 (-0.106, -0.044)** | **-0.075 (-0.106, -0.044)** | **-0.045 (-0.077, -0.013)** | **-0.074 (-0.105, -0.042)** | **-0.074 (-0.107, -0.041)** | - |
| Language | Sleep**↓** | - | -0.002 (-0.006, 0.002) | **0.021 (0.010, 0.031)** | 0.002 (-0.002, 0.005) | 0.001 (-0.005, 0.006) | **0.034 (0.009, 0.059)** |
|  | TV Watching**↓** | 0.002 (-0.002, 0.007) | - | **0.023 (0.014, 0.031)** | **0.004 (0.001, 0.006)** | 0.003 (-0.001, 0.007) | **0.036 (0.010, 0.061)** |
|  | Use Computer**↓** | N/A | N/A | - | N/A | N/A | N/A |
|  | Other SB**↓** | -0.002 (-0.005, 0.002) | **-0.004 (-0.006, -0.001)** | **0.019 (0.010, 0.028)** | - | -0.001 (-0.004, 0.002) | **0.032 (0.006, 0.058)** |
|  | LPA**↓** | -0.001 (-0.006, 0.005) | -0.003 (-0.007, 0.001) | **0.020 (0.012, 0.028)** | 0.001 (-0.002, 0.004) | - | **0.033 (0.005, 0.061)** |
|  | MVPA**↓** | **-0.059 (-0.104, -0.014)** | **-0.061 (-0.106, -0.016)** | -0.039 (-0.086, 0.009) | **-0.058 (-0.103, -0.012)** | **-0.059 (-0.106, -0.011)** | - |
| Execution Function | Sleep**↓** | - | -0.0001 (-0.003, 0.003) | **0.033 (0.026, 0.040)** | 0.001 (-0.002, 0.003) | -0.004 (-0.009, 0.0002) | **0.067 (0.049, 0.084)** |
|  | TV Watching**↓** | 0.0001 (-0.003, 0.003) | - | **0.033 (0.025, 0.041)** | 0.001 (-0.001, 0.002) | **-0.004 (-0.007, -0.001)** | **0.067 (0.048, 0.086)** |
|  | Use Computer**↓** | N/A | N/A | - | N/A | N/A | N/A |
|  | Other SB**↓** | -0.001 (-0.003, 0.002) | -0.001 (-0.002, 0.001) | **0.032 (0.024, 0.040)** | - | **-0.005 (-0.008, -0.002)** | **0.066 (0.047, 0.085)** |
|  | LPA**↓** | 0.004 (-0.0001, 0.009) | **0.004 (0.001, 0.007)** | **0.037 (0.029, 0.045)** | **0.005 (0.002, 0.008)** | - | **0.071 (0.051, 0.091)** |
|  | MVPA**↓** | **-0.118 (-0.150, -0.086)** | **-0.118 (-0.151, -0.085)** | **-0.085 (-0.118, -0.053)** | **-0.117 (-0.150, -0.084)** | **-0.122 (-0.157, -0.088)** | - |
| Global Cognition | Sleep**↓** | - | -0.001 (-0.004, 0.002) | **0.034 (0.027, 0.041)** | 0.002 (-0.001, 0.004) | -0.001 (-0.005, 0.003) | **0.059 (0.040, 0.077)** |
|  | TV Watching**↓** | 0.001 (-0.002, 0.004) | - | **0.035 (0.029, 0.042)** | **0.003 (0.001, 0.004)** | 0.0001 (-0.003, 0.003) | **0.060 (0.039, 0.080)** |
|  | Use Computer**↓** | N/A | N/A | - | N/A | N/A | N/A |
|  | Other SB**↓** | -0.002 (-0.004, 0.001) | -0.003 (-0.004, -0.001) | **0.033 (0.026, 0.039)** | - | **-0.003 (-0.005, -0.0004)** | **0.057 (0.037, 0.077)** |
|  | LPA**↓** | 0.001 (-0.003, 0.005) | 0.00005 (-0.003, 0.003) | **0.035 (0.029, 0.042)** | **0.003 (0.0003, 0.005)** | - | **0.060 (0.038, 0.082)** |
|  | MVPA**↓** | **-0.103 (-0.137, -0.069)** | **-0.104 (-0.140, -0.069)** | **-0.069 (-0.105, -0.033)** | **-0.102 (-0.137, -0.066)** | **-0.104 (-0.141, -0.067)** | - |

*Note.* SB: sedentary behavior; LPA: light physical activity; MVPA: moderate and vigorous physical activity. The model was weighted based on the complex sampling design of NHANES and adjusted for age, sex, education level, marriage, ethnicity, obesity, smoke status, excessive drinking, hypertension, diabetes, and physical function limitations. The significant movement results were bold. Due to the non-negativity constraint of compositional data, isotemporal substitution of time from computer use to other behaviors was not computed, as the geometric mean of computer use (7.90 min/day) is less than the 10-minute reallocation increment. Reallocations of time to computer use from other behaviors remain interpretable and are presented.

**Table S3.** **Predicted changes of cognitive function for reallocating 10-min between one and the remaining behaviors proportionally**

| Outcome | Movements | Reallocate 10 min from column movement into remaining behaviors | Reallocate 10 min from remaining behaviors into column movement |
| --- | --- | --- | --- |
| Memory | Sleep | 0.002 (-0.001, 0.005) | -0.002 (-0.005, 0.001) |
|  | TV Watching | 0.002 (-0.0002, 0.004) | -0.002 (-0.004, 0.0002) |
|  | Use Computer | N/A | **0.029 (0.018, 0.040)** |
|  | Other SB | -0.0002 (-0.002, 0.001) | 0.0002 (-0.001, 0.001) |
|  | LPA | 0.0004 (-0.003, 0.004) | -0.0004 (-0.004, 0.003) |
|  | MVPA | **-0.074 (-0.106, -0.043)** | **0.042 (0.024, 0.060)** |
| Language | Sleep | 0.001 (-0.003, 0.005) | -0.001 (-0.005, 0.003) |
|  | TV Watching | **0.003 (0.0004, 0.006)** | **-0.003 (-0.006, -0.0004)** |
|  | Use Computer | N/A | **0.020 (0.011, 0.029)** |
|  | Other SB | -0.001 (-0.003, 0.0007) | 0.001 (-0.001, 0.002) |
|  | LPA | 0.0001 (-0.005, 0.005) | -0.0001 (-0.005, 0.004) |
|  | MVPA | **-0.059 (-0.105, -0.013)** | **0.034 (0.007, 0.060)** |
| Executive Function | Sleep | -0.00001 (-0.003, 0.003) | 0.00001 (-0.003, 0.003) |
|  | TV Watching | 0.0002 (-0.002, 0.002) | -0.0002 (-0.002, 0.002) |
|  | Use Computer | N/A | **0.033 (0.026, 0.040)** |
|  | Other SB | -0.001 (-0.002, 0.001) | 0.001 (-0.001, 0.002) |
|  | LPA | **0.006 (0.002, 0.010)** | **-0.006 (-0.010, -0.002)** |
|  | MVPA | **-0.119 (-0.152, -0.086)** | **0.068 (0.049, 0.087)** |
| Global Cognition | Sleep | 0.001 (-0.002, 0.004) | -0.001 (-0.004, 0.002) |
|  | TV Watching | **0.002 (0.00003, 0.005)** | **-0.002 (-0.004, -0.00003)** |
|  | Use Computer | N/A | **0.034 (0.027, 0.040)** |
|  | Other SB | -0.001 (-0.002, 0.0003) | 0.001 (-0.0003, 0.002) |
|  | LPA | 0.003 (-0.001, 0.006) | -0.003 (-0.006, 0.001) |
|  | MVPA | **-0.103 (-0.139, -0.068)** | **0.059 (0.039, 0.079)** |

*Note.* SB: sedentary behavior; LPA: light physical activity; MVPA: moderate and vigorous physical activity. The model was weighted based on the complex sampling design of NHANES and adjusted for age, sex, education level, marriage, ethnicity, obesity, smoke status, excessive drinking, hypertension, diabetes, and physical function limitations. The significant movement results were bold. Due to the non-negativity constraint of compositional data, isotemporal substitution of time from computer use to other behaviors was not computed, as the geometric mean of computer use (7.90 min/day) is less than the 10-minute reallocation increment. Reallocations of time to computer use from other behaviors remain interpretable and are presented.
